# Reliability of Panoramic Ultrasonography in Assessing Pectoralis Minor Morphology: An Intra- and Inter-Rater Study Among Asymptomatic Adults

**DOI:** 10.3390/jfmk11030283

**Published:** 2026-07-21

**Authors:** Rijal Rijal, Huei-Ming Chai, Jiu-Jenq Lin, Shan Fam

**Affiliations:** 1 School and Graduate Institute of Physical Therapy, College of Medicine, National Taiwan University, Taipei City 10055, Taiwan; jiujlin@ntu.edu.tw (J.-J.L.); r10428015@ntu.edu.tw (S.F.); 2 Physiotherapy Department, Faculty of Nursing, Hasanuddin University, Makassar 90245, Indonesia; 3 Physical Therapy Center, National Taiwan University Hospital, Taipei 100002, Taiwan; 4 Department of Athletic Performance, National Taiwan Normal University, Taipei 11677, Taiwan

**Keywords:** panoramic ultrasonography, pectoralis minor morphologies, rounded shoulder posture

## Abstract

**Objectives**: This study aimed to examine the intra- and inter-rater reliabilities of panoramic ultrasonographic measurements of the pectoralis minor length (Pm_L), pectoralis minor thickness (Pm_T), and pectoralis minor cross-sectional area (Pm_CSA). **Methods**: Twenty young male participants aged 19–35 years were recruited for this study. All participants had asymptomatic rounded shoulder posture (RSP), with a mean acromion-to-table distance of 59.316 ± 9.631 mm. Longitudinal panoramic scans of the Pm of the dominant arm were acquired in three trials of four measurements for all participants. The first two measurements were examined by the same rater on the same day, and the third measurement was performed by a different rater. The same procedure was carried out by the first rater after 30 days for inter-day reliability. Intraclass correlation coefficients (ICCs), standard errors of measurement (SEMs), and minimal detectable changes (MDCs) were calculated to estimate the reliabilities. **Results**: The intra- and inter-rater reliabilities of Pm_L, Pm_T and Pm_CSA were excellent (all ICC > 0.932; all SEM < 7.4%; all MDC < 20%). Bland–Altman plots demonstrated good agreement with nearly zero mean differences between measurements. **Conclusions**: Using panoramic ultrasonography to measure Pm_L, Pm_T and Pm_CSA may be considered a reliable method with excellent inter-day, intra- or inter-rater reliabilities.

## 1. Introduction

The acromion-to-table distance (ATD) has been introduced as a measurement for the tightness of the pectoralis minor (Pm) in the past several decades, but an increased ATD can be affected not just by the length of the Pm. The tightness of the Pm is one of the etiological factors of RSP [1,2]. RSP has been defined as the forward displacement of the acromion process of the scapula relative to the spinous process of the seventh cervical vertebra. The significantly increasing prevalence of RSP is noted in sedentary workers in modern society [3] or athletes with upper extremity sports [4]. Since ATD has been introduced as a measurement of the tightness of the Pm, it has been regarded as the most widely used index for RSP in the past several decades. However, multiple etiological factors may contribute to RSP. Clinically, RSP is evaluated using observation or inspection with a plumb line [5], but observations or inspections of an individual might be subject to the observer’s bias and fail to demonstrate objective results. Therefore, they postulated a quantitative ATD test to determine RSP by measuring the distance from the posterior border of the acromion process to the table as the individual to be tested was lying in the supine position. They regarded the ATD as an objective measurement of Pm_L. However, the ATD is an indirect measurement of Pm_L and an increased ATD may not be simply attributed to the tightness of the Pm [6,7]. Weakness of the scapular muscles may also change the ATD [4]. Consequently, a reliable alternative to directly measure the Pm morphology is crucial for assessing the effectiveness of any given intervention for individuals with tightness of the Pm.

Although the Vernier caliper or tape measure methods may measure Pm_L directly, the results of their measurements are inconsistent with those from cadaver studies. Subsequent direct measurements were performed using various tools to measure Pm_L. The high reliability of the measurement of Pm_L using either the Vernier caliper or tape measure methods (ICC = 0.82–0.87) was demonstrated [8]. Measurements of Pm_L using a caliper or tape measure on human bodies were 15.6 and 16.1 cm, respectively. However, the Pm_L was 12.6 cm when the cadavers were examined, which was far from the in vivo measurements. It was also argued that the in vitro measurement has an excellent intraclass correlation coefficient (0.96) and an SEM of approximately 0.66. Although the Pm_L measurement represents Pm morphology, in vivo measurements were inconsistent with cadaver in vitro measurements [8]. Moreover, physical assessments such as calipers and tape measures cannot measure Pm_T and Pm_CSA. Clinically, the whole muscle morphology of the Pm, including Pm_L, Pm_T, and Pm_CSA, needs to be evaluated. For example, the location and size of the tear of the Pm are necessary to be measured for clinical decision-making. Therefore, the need for imaging techniques to evaluate the Pm morphologies without invasive procedures arose spontaneously.

Magnetic resonance imaging (MRI) is widely regarded as a reference standard for evaluating muscle morphology. However, its cost, accessibility, and operational requirements may limit its routine clinical use. Ultrasonography offers a practical, non-invasive, and accessible alternative for assessing muscle morphology. Nevertheless, the validity of panoramic ultrasonography for evaluating pectoralis minor morphology relative to MRI has not yet been established and warrants further investigation. Magnetic resonance imaging (MRI) is widely regarded as a reference imaging modality for evaluating skeletal muscle morphology because of its high spatial resolution and ability to visualize soft tissues non-invasively [8]. However, MRI causes high costs in operating and maintaining the machine and is difficult to access, resulting in not being widely available [8]. Ultrasonography (USI) is a safe and effective tool that can generate high-quality images to depict muscle morphologies compared to MRI [9]. B-mode USI provides a 2D planar image of the scanned tissue, in which the sound-reflecting interfaces of the muscle, fascia, and perimysium are delineated as bright structures [10]. They also demonstrated high reliability in measuring the thickness and cross-sectional area of the supraspinatus muscle, with an ICC of 0.91 and 0.90, respectively. B-mode USI has high reliability in measuring muscle morphology, but ultrasonography has limitations in measuring muscle morphology that exceeds the width of the USI probe.

Currently, panoramic USI provides an extended field of muscle view morphology to overcome the limitations of B-mode USI in observing the whole muscle status. The ability of B-mode USI to assess muscle thickness has been established, but B-mode USI cannot present the whole status of the muscle if the muscle length is greater than the width of the ultrasonographic probe [11]. Most USI probes worldwide are designed with a width of 3.8–10 cm [10]. B-mode USI has limitations in observing anatomical structures with lengths greater than the probe width. As a result, this method cannot measure the entire status to identify other morphologies, such as muscle length and cross-sectional area [10]. Another reason is that single transverse imaging and panoramic USI imaging are comparable [12]. However, they found that panoramic USI has a coefficient of variation (2.78%) lower than single transverse (3.28%). The results indicated that the panoramic USI data were more stable and consistent than the single transverse ultrasonographic data. Therefore, panoramic ultrasonography is a solution for structures whose size is larger than an ultrasound probe and may be an alternative for evaluating Pm morphologies.

Recently, panoramic USI has been used to observe muscle morphologies, but no study has examined the Pm muscle morphologies. Panoramic ultrasonographic captures continuous combined images as the ultrasonographic probe moves over the outer layer of an anatomical structure [10,13]. Therefore, panoramic USI is a solution for measuring the entire status of the muscle [13,14,15]. However, less is known about assessing deep scapular muscles, especially for Pm morphologies, by applying panoramic USI. Therefore, examination of the Pm muscle morphologies, including muscle length, thickness, and cross-sectional area, is necessary for individuals with RSP. Thus, this study aimed to determine the intra-rater within-day, intra-rater inter-day, and inter-rater reliabilities of panoramic ultrasonographic techniques in measuring the muscle morphologies of the Pm muscle in terms of the ICC, SEM, MDC, and Bland–Altman plot.

## 2. Materials and Methods

Twenty healthy young male volunteers aged 19–35 years (mean age 22.9 ± 3.8 years) participated in this study. Convenience sampling was used to recruit participants via public advertisements on Facebook and Instagram. The inclusion criteria were healthy asymptomatic males aged 18–65 years with rounded shoulder posture (ATD > 2.6 cm). Since exposure to the chest was required during the experiment, only male participants were recruited for this study. Participants were excluded if they presented any of the following conditions: severe shoulder pain or limited range of motion in the past 3 months; any history of fracture, subluxation, or dislocation at the upper extremity; history of cervical or shoulder surgery; or any systemic, neurological, or musculoskeletal disease. All participants were screened for eligibility first. The dominant arm, which was the side to be tested, was evaluated in each participant by asking them to write on paper to determine the dominant side. The participants were subsequently informed of the purposes and procedures of this study and signed a written informed consent form before the experiment. The study protocol was approved by the Research Ethics Committees of the National Taiwan University Hospital (issued number 202401150RINE).

An a priori sample size calculation was performed using G*Power version 3.1.9.6 (Heinrich-Heine University, Düsseldorf, Germany). The calculation was based on pectoralis minor thickness values, which served as the primary outcome measure because it was the most comparable ultrasonographic variable available in previous research [16]. Using a significance level of α = 0.05 and a statistical power of 80% (1 − β = 0.80), the minimum required sample size was estimated to be 15 participants. To increase the precision of the reliability estimates and account for potential exclusions or missing data, 20 participants were ultimately recruited for this study.

This study aimed to determine the within-day intra-rater, between-day intra-rater, and inter-rater reliabilities. All ultrasonographic imaging procedures were performed by 2 raters. The primary rater (RR) was a physical therapist with 20 years of experience in musculoskeletal physical therapy, while the second rater (SF) was a physical therapist with 3 years of experience in sports physical therapy. Both had more than 3 years of experience in USI. Two raters practiced the entire measurement procedure for 3 months to ensure the quality of the panoramic ultrasonographic images. Intra-rater reliability was defined as the 2 measurements taken by the primary rater either at different sessions on the same day within an hour (within-day intra-rater reliability) or on different days with a 30-day interval (between-day intra-rater reliability). Inter-rater reliability was determined by the difference between the primary and secondary raters. The raters and participants were blinded to the reliability results during the experiment.

**Figure 1:**
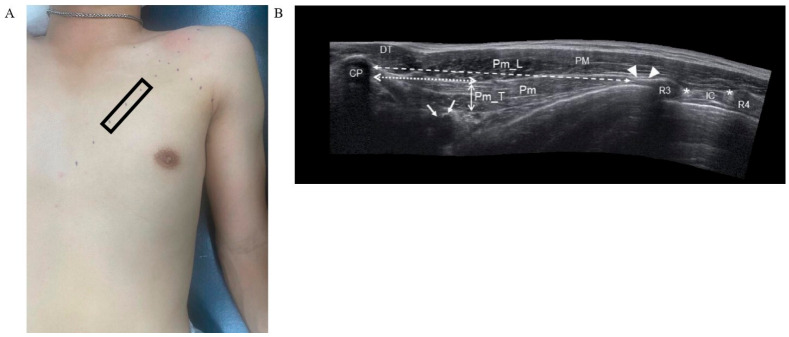
Placement of the probe and measurement on a panoramic image for the length and thickness of the Pectoralis minor (**A**). Panoramic ultrasonographic image of Pm_L and Pm_T (**B**). Pm_L (dash line); 1/3 of Pm_L (round dot line); Pm_T (solid line); CP, coracoid process; DT, deltoid; PM, pectoralis major; IC, intercostalis; R3, third rib; R4, fourth rib; costochondral junctions (head arrows); axillary artery (arrows); costal cartilage (asterisk). The black box indicates the ultrasonographic probe position.

All Pm morphologies in each condition were measured by 2 raters using panoramic USI. The USI system (SonoAce R7, Samsung Medison Co., Ltd., Seoul, Republic of Korea) was equipped with a 5–12 MHz linear probe, and the image resolution was 0.1 mm. The depth of each image was set to 60 mm for collecting the whole thickness of the Pm. The focus was fixed at 30–50 mm for better resolution of the Pm muscle images. All ultrasonographic images of the Pm were acquired when the participants were lying on a plinth in the supine and relaxed position, supinating their forearms with their hands facing upward, and placing their arms beside their body. The rater used the 2D-mode USI to identify the superior medial border of the coracoid process of the scapula and the inferior aspect of the 4th rib first and marked these two bony landmarks using a surgical marker. A straight line to connect these two markers was drawn on the skin, indicating the longitudinal axis of the Pm (Figure 1A).

**Figure 2:**
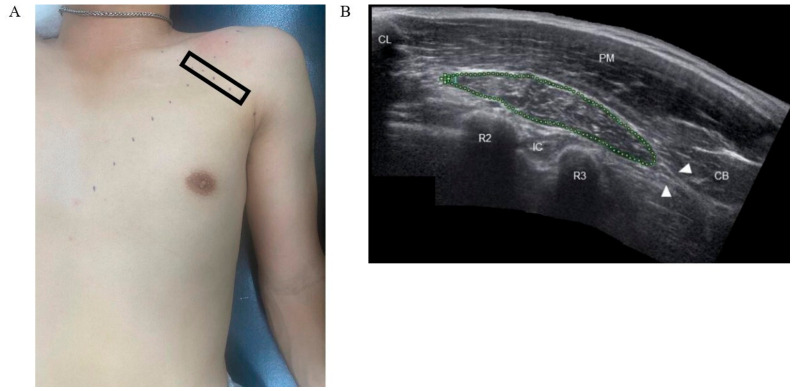
Placement of the probe and measurement on a panoramic image for the cross-sectional area of the Pm (**A**). Panoramic of pectoralis minor (Pm) cross-sectional area (**B**). PM, pectoralis major; CB, coracobrachialis; IC, intercostalis; Pm_CSA, pectoralis minor cross-sectional area; CL, clavicula; FAX, fascia axillaris (head arrows); R2, second rib; R3, third rib. The black box indicates the ultrasonographic probe position. The green dotted outline indicates the Pm_CSA.

Subsequently, three trials of two panoramic ultrasonographic images were acquired to measure Pm_L, Pm_T, and Pm_CSA using the following methods. The raters placed the ultrasonographic probe on the lateral end of the longitudinal axis line to obtain panoramic ultrasonographic images of Pm_L. The participant was asked to exhale, then inhale optimally, and continue exhaling again. After the second exhalation, the rater asked the participant to hold their breath at the end of expiration to reduce the influence of respiration and contraction of the Pm during image acquisition. Meanwhile, the rater slid the probe along the longitudinal-axis line to obtain a panoramic image. Pm_L was measured from the superior medial border of the coracoid process to the medial end of the muscle by identifying the tendon location on the panoramic image (Figure 1B). Then, the rater marked a point at the lateral one-third of the longitudinal-axis line on the image, and made another line perpendicular to this point for measurement of Pm_T (Figure 1B). The second panoramic ultrasonographic image was obtained to measure Pm_CSA. A cross line on the skin surface from the clavicle to the anterior axillary line was drawn, indicating the transverse axis of the Pm (Figure 2A). Subsequently, another panoramic ultrasonographic image was obtained (Figure 2B). Three trials of image acquisition were conducted for each muscle morphology to achieve the highest possible quality. All data from the averaged 3 trials were used for statistical analyses.

All statistical analyses were performed using SPSS version 20.0 software (SPSS for Windows; SPSS Inc., Chicago, IL, USA). The data were initially examined for normality using the Shapiro–Wilk (S-W) test. The statistical significance threshold was set at *p* < 0.05. If the *p*-value was >0.05, the null hypothesis was accepted, indicating that the dataset had a high possibility of being a normal distribution. Means and standard deviations were used for the descriptive analysis of continuous variables, while frequencies and percentages were used for discrete variables.

The ICC values and 95% confidence interval (95% CI) were employed to evaluate both intra- and inter-rater reliability. The ICC (3, 3) and ICC (2, 3) models were selected to determine the intra-rater and inter-rater reliabilities, respectively. Therefore, in the SPSS program, a model of two-way mixed effects with consistency and multiple raters/measurements was used to calculate the ICC value for intra-rater reliability. In contrast, a two-way random effects model with absolute agreement and multiple raters/measurements was chosen to estimate the ICC values for inter-rater reliability. The ICC values were classified as follows: poor (<0.50), moderate (0.50–0.75), good (0.75–0.90), and excellent (>0.90) [17]. The 95% CI of the ICC estimates was calculated for each condition.

The SEM for the absolute reliability was calculated by

$$\mathrm{SEM}=\mathrm{SD}\times \sqrt{\left(1-ICC\right)}\;\mathrm{and}$$

$$SEM\%=\frac{SEM}{Mean}\times 100$$

where SEM = standard error of measurement; SD = standard deviation; ICC = intraclass correlation coefficient [18].

MDC is a minimal disparity between sessions that may be recognized as genuine alterations for treatment purposes.

MDC = (SEM × 1.96 × √ 2) and

$$MDC\%=\frac{MDC}{Mean}\times 100,$$

where MDC = minimal detectable change; SEM = standard error of measurement [18].

Generally, SEM% values below 10% and MDC% values below 30% are considered acceptable for SEM and MDC [19].

Agreement between repeated measurements was additionally assessed using Bland–Altman analysis. For each comparison, the mean difference (bias) and the 95% limits of agreement (mean difference ± 1.96 × standard deviation of the differences) were calculated and reported [20].

## 3. Results

All 20 participants completed the imaging procedures successfully. No ultrasonographic images were excluded because of inadequate image quality, incomplete visualization of the pectoralis minor muscle, or measurement errors. Therefore, the image acquisition success rate was 100% among the enrolled participants. Their basic information is listed in Table 1. All participants were young and active people, who engaged in regular exercise every week and used a computer in daily activities. Seventy-five percent of participants (15/20) spent more than 4 h per day in front of a computer. They were all RSP based on the definition of ATD greater than 2.6 cm, but they were asymptomatic because the shoulder pain and disability index (SPADI) score was less than 45%. For the normality tests of all continuous variables, the *p*-values of all S-W tests were greater than 0.05, revealing that the demographic and baseline outcome variables for this study might be normally distributed.

Panoramic ultrasonographic measurements of the Pm morphology variables demonstrated excellent agreement between the first and second measurements, either within the same day, between 1-month intervals, or between two raters. The group means and standard deviations of all panoramic ultrasonographic measurements are presented in Table 2. The ICC values for the intra- and inter-rater reliabilities were all greater than 0.933 for the panoramic ultrasonographic measurements of the Pm morphology variables (Table 1, Table 2, Table 3 and Table 4), indicating excellent reliabilities. All SEM values of panoramic ultrasonographic muscle morphology measurements ranged from 0.7 to 5.9%, indicating that the panoramic ultrasonographic measurements were reliable and precise. All MDC values ranged from 2.0 to 16.4%, indicating that the true changes after any given intervention must be greater than these MDC values. All SEM values were less than 10%, and all MDC values less than 30% showed a high level of accuracy for these measurements.

**Figure 3:**
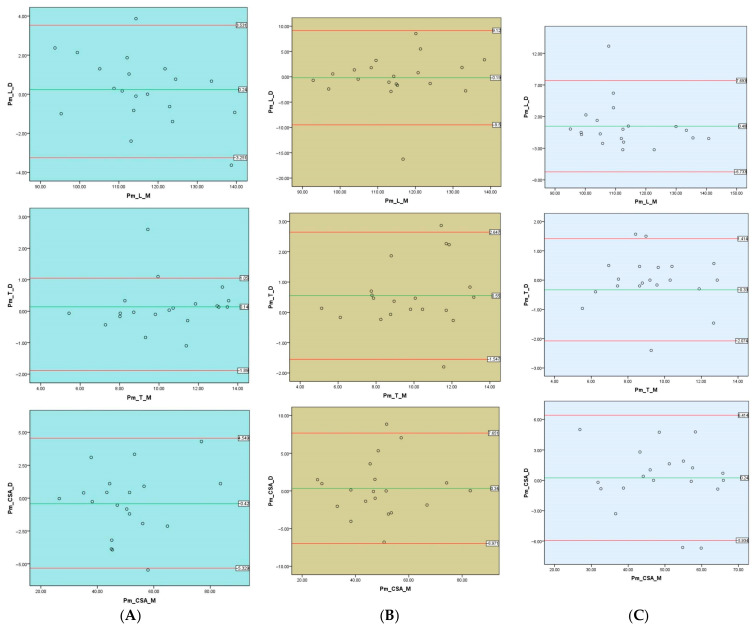
Bland–Altman plots for the panoramic USI to measure relative changes in the Pm_L, Pm_T, and Pm_CSA between the first and second measurements in within-day (**A**); between the first and second raters in within-day (**B**); between the first and second measurements after 30 days (**C**). The green horizontal line represents the mean difference (bias), the red horizontal lines represent the upper and lower limits of agreement (mean difference ± 1.96 SD).

Bland–Altman plots for all panoramic ultrasonographic measurements also demonstrated good agreement, with nearly zero mean differences. It showed good agreement in results of measurements between two sessions within the same day (Figure 3A) or between 1-month intervals for the same rater (Figure 3C). The mean differences were close to zero (0.1–0.4 mm). This was also the case for the data between two different raters, and the mean difference was also almost null (0.1–0.5 mm) (Figure 3B). The results indicated that panoramic ultrasonographic measurements consistently produced similar measurements of the Pm morphology variables. These measurements are reliable and sufficiently sensitive to detect changes in clinical practice or research.

Bland–Altman analysis demonstrated good agreement between measurements. For Pm_L, the mean difference between the first and second measurements was approximately 0.02 mm, with 95% limits of agreement ranging from −0.31 to 0.35 mm. For Pm_T, the mean difference was approximately 0.01 mm, with limits of agreement ranging from −0.16 to 0.13 mm. For Pm_CSA, the mean difference was approximately −0.04 mm^2^, with limits of agreement ranging from −0.54 to 0.45 mm^2^. Similar results were observed for both inter-rater and between-day comparisons, with mean differences close to zero and relatively narrow limits of agreement, indicating minimal systematic bias.

## 4. Discussion

This study examined the ability of panoramic USI to consistently reproduce the muscle morphology values of the Pm muscles in asymptomatic male participants. To the best of our knowledge, this study is the first to utilize panoramic USI to assess the muscle morphology of the Pm. These findings showed that the ICC was excellent in using panoramic USI to measure muscle morphologies, including muscle length, thickness, and cross-sectional area. Regarding intra- and inter-rater reliability, the SEM of the two measurement points was incredibly small at both points of interest.

According to the ICC value, the measurement of the pectoralis minor length using panoramic USI exhibited greater reliability than physical assessments because of the limitations of palpation skills and the presence of soft tissues over the Pm muscle. The reliability of panoramic ultrasonographic measurement of pectoralis minor length was excellent, with ICC above 0.963 for intra- and inter-rater reliability. These findings align with those of a previous study that demonstrated excellent reliability in vitro using cadavers [8]. He also reported that measuring the pectoralis minor length using a Vernier caliper or tape had an ICC level in a good category of 0.870 and 0.860, respectively. The quantity of subcutaneous tissue over the anatomical landmarks was the main reason for palpation error in both cadavers and live participants [21]. Therefore, the inter-rater measurement of the pectoralis minor length was categorized as poor, with a value of 0.370 [21]. Both examiners noted that a significant amount of soft tissue over the Pm in females was an obstacle in measuring the pectoralis minor length. Consequently, the raters’ ability to identify bony landmarks was inconsistent, resulting in a low level of reliability.

Furthermore, the reliability of measuring Pm thickness using panoramic USI was excellent, and the 95% CI was narrower than that of B-mode USI. The current study reported that panoramic ultrasonographic measurement of the pectoralis minor thickness was an excellent category with an ICC above 0.933 for intra- and inter-rater reliability. The findings align with an earlier study that revealed the excellent intra- and inter-rater reliability of B-mode USI using a linear transducer for measuring the pectoralis minor thickness [16]. Although previous research has also demonstrated excellent reliability, the ICC values and confidence intervals observed in the present study indicate strong agreement of panoramic ultrasonographic measurements of pectoralis minor morphology. Although these estimates appear favorable when compared with previous reports, methodological and population differences between studies preclude definitive conclusions regarding the relative performance of different ultrasonographic techniques. A direct head-to-head comparison study would be required to determine whether panoramic ultrasonography offers advantages over conventional B-mode ultrasonography. The 95% CI of the current study was from 0.928 to 0.998, while the previous study started from 0.875 to 0.985 [22]. The reliability estimates observed in the present study were comparable to those reported in previous studies using conventional ultrasonographic techniques. However, direct comparisons between studies should be interpreted cautiously because ICC values and confidence intervals are influenced by differences in participant characteristics, sample variability, examiner experience, imaging protocols, and statistical procedures. Therefore, the current findings should not be interpreted as evidence of superiority of panoramic ultrasonography over other ultrasonographic methods.

Determining one-third of the Pm_L and bony landmark palpation may explain the reason for the narrower confidence interval observed in the current study compared to previous studies. The measurement of muscle thickness at one-third of the Pm_L was conducted on the skin surface [16]. They measured one-third of the Pm_L from the inferior border of the acromion to the inferior border of the fourth rib. In contrast, the current study utilized panoramic USI to facilitate the direct measurement of the Pm muscle, as the entire muscle image was visible on the screen. Therefore, it allowed the raters to measure one-third of the Pm_L precisely to determine the thickest part of the Pm. Another reason is that the previous research required an accurate method for palpating bony landmarks, especially the coracoid process and the fourth rib [16]. However, they found that both raters had difficulty palpating the bony landmarks due to the subcutaneous tissue over the anatomical structure.

In addition, this study also demonstrated the high reproducibility of panoramic USI in measuring the pectoralis minor cross-sectional area, and the accuracy of ultrasonographic images can be influenced by BMI and the rater’s skill and experience. This is the first time this method has been used in a study to measure the pectoralis minor cross-sectional area using panoramic USI with consistent measurements above 0.983 for intra- and inter-rater reliability. The study results align with a previous study, where the CSA of the pectoralis major was classified as having excellent reliability (0.939) [22]. The previous study involved some participants who were categorized as overweight and obese and the authors found that the raters experienced difficulty in identifying the CSA due to the fat layers, especially at the lower border of the muscle [23]. The adverse effects of fat layers on ultrasonographic images were increased due to the adipose tissue distorting the ultrasonographic images [23]. A different study also compared the CSA of the pectoralis major measurement between trained versus novice individuals [22]. They demonstrated that the SEM was significantly smaller for the trained operator compared to the novice operator, with a SEM of 0.25 cm^2^ and 0.66 cm^2^, respectively. Panoramic ultrasonographic measurements may also depend on raters’ probe alignment, pressure, and movement velocity during image acquisition [23].

In summary, this study provides solid evidence that Pm morphological measurements using panoramic USI are highly reliable, and several advantages have been identified when comparing the findings with those of previous studies. This study is the first to measure Pm morphology using panoramic USI. The results showed that the reliabilities on the Pm morphologies were above 0.900. All SEMs were below 10%, and all MDCs were below 30%. The Bland–Altman plots showed good agreement between the inter-day and intra-rater reliability between the two measurement sessions and between the two raters, with a mean difference close to zero (0.1–0.5 mm). Outliers were present at all points in the intra- and inter-rater reliability assessments. The current study applied panoramic ultrasonographic measurements to participants in the standard testing position, stabilizing the upper extremity in the lying position. The interval was about 30 min, more than that in the previous study. According to the previous and present findings, the measurements of the Pm morphologies were performed with high reliability. From a clinical perspective, panoramic ultrasonography offers several practical advantages, including portability, non-invasiveness, real-time image acquisition, and lower cost compared with advanced imaging modalities. In the present study, image acquisition was performed by physical therapists with more than three years of musculoskeletal ultrasonography experience who underwent an additional three-month training period to standardize the scanning procedure. Therefore, successful implementation of this technique may require adequate operator training and familiarity with musculoskeletal anatomy and panoramic scanning techniques. The complete examination required approximately 8–10 min per participant, including landmark identification, image acquisition, and offline image analysis. This would substantially strengthen this study because it demonstrates not only that the method is reliable but also that it is practically implementable in clinical settings.

Although the present study demonstrated excellent intra-rater and inter-rater reliability of panoramic ultrasonography for measuring pectoralis minor morphology, reliability should not be interpreted as evidence of validity. The current study did not compare panoramic ultrasonographic measurements with MRI or any other reference imaging modality. Therefore, the accuracy of panoramic ultrasonography in quantifying pectoralis minor morphology remains to be established. Future studies should investigate criterion validity by directly comparing panoramic ultrasonographic measurements with MRI-derived measurements.

Several limitations should be considered when interpreting the findings of this study. First, it included only young asymptomatic male participants with rounded shoulder posture; therefore, the reliability estimates may not be directly applicable to females, older adults, symptomatic individuals, or athletic populations. Differences in muscle morphology, body composition, pain status, activity level, and anatomical characteristics may influence ultrasonographic image acquisition and measurement reliability. In particular, sex-related differences in body composition, including the distribution and thickness of subcutaneous adipose tissue over the anterior chest wall, may affect image quality, anatomical landmark identification, and measurement accuracy. An additional limitation of this study is that the reliability estimates were derived from a single cohort of young asymptomatic male participants evaluated in one research setting. Consequently, the findings have not been externally confirmed in independent populations or clinical environments. Differences in participant characteristics, examiner expertise, equipment, and imaging protocols may influence reliability estimates. Therefore, future studies should investigate the reproducibility of these findings across multiple centers, broader populations, and clinicians with varying levels of ultrasonography experience to strengthen the external validity of panoramic ultrasonographic assessment of pectoralis minor morphology.

Moreover, the reliability estimates reported in this study were obtained by experienced examiners who underwent protocol-specific training before data collection. Therefore, the results may not fully reflect the reliability achievable by novice operators or clinicians with limited experience in panoramic ultrasonography. Further studies should investigate the learning curve and minimum training requirements needed to achieve acceptable measurement reliability.

## 5. Conclusions

This study is the first to investigate the reliability of panoramic ultrasonography for assessing pectoralis minor morphology, including muscle length, thickness, and cross-sectional area, in young asymptomatic males with rounded shoulder posture. The results demonstrated excellent within-day and between-day intra-rater reliability as well as excellent inter-rater reliability, with high ICC values, low standard errors of measurement, and acceptable minimal detectable changes. These findings suggest that panoramic ultrasonography is a consistent and reproducible method for evaluating pectoralis minor morphology in this population.

From a clinical perspective, panoramic ultrasonography may provide a practical, non-invasive, and accessible approach for monitoring the morphological characteristics of the pectoralis minor muscle. However, the findings should be interpreted within the context of the study population and should not be generalized to females, older adults, athletes, or individuals with musculoskeletal disorders. Furthermore, because this study assessed reliability rather than validity, future studies should investigate the accuracy of panoramic ultrasonography against reference imaging modalities and evaluate its feasibility in routine clinical practice.

## Figures and Tables

**Table 1:** Participants’ demographic and baseline data of the reliability study.

Continuous Variables	Mean ± SD	Range	S-W Test Statistics
Age (year)	22.9 ± 3.8	19–35	0.471
Body height (cm)	174.62 ± 6.07	167.5–187.0	0.767
Body weight (kg)	70.50 ± 9.86	56.0–95.5	0.706
BMI (kg/m^2^)	23.10 ± 2.58	17.6–28.6	0.558
PA (times/week)	2.9 ± 1.3	1–6	0.292
PA duration (min/session)	91.5 ± 24.7	60–120	0.062
ATD (mm)	59.316 ± 9.631	35.61–71.19	0.107
Pm_L (mm)	115.8 ± 12.6	93–139	0.514
Pm_T (mm)	10.3 ± 2.2	5–13	0.486
Pm_CSA (mm^2^)	50.5 ± 13.4	26–83	0.236
Ordinal variable	Median	IQR	
SPADI (%)	1.9	0–6.7	
Categorical variables	Frequency	Percentage (%)	
Computer use (h)			
➢ <4	5	25	
➢ 4–8	9	45	
➢ >8	6	30	
Dominant arm			
➢ R	19	95	
➢ L	1	5	

Continuous variables are expressed as mean ± SD, discrete variables as median (IQR), and categorical variables as frequency (%). Abbreviations: ATD, acromion-to-table distance; BMI, body mass index; IQR, interquartile range; L, left; PA, physical activity; Pm_L, pectoralis minor length; Pm_T, pectoralis minor thickness; Pm_CSA, pectoralis minor cross-sectional area; R, right; S-W test, Shapiro–Wilk test; SD, standard deviation; SPADI, shoulder pain and disability index.

**Table 2:** Within-day intra-rater reliability in the reliability study.

Outcomes	ICC (95% CI)	SEM (mm)	SEM (%)	MDC (mm)	MDC (%)
Pm_L (mm)	0.995 (0.989–0.998)	0.83	0.7	2.30	2.0
Pm_T (mm)	0.974 (0.934–0.990)	0.37	3.6	1.02	9.9
Pm_CSA (mm^2^)	0.991 (0.979–0.997)	1.28	2.5	3.54	7.0

Abbreviations: 95% CI, 95% confidence interval of ICC; ATD, acromion-to-table distance; ICC, intraclass correlation coefficient; MDC, minimal detectable change; Pm_L, pectoralis minor length; Pm_T, pectoralis minor thickness; Pm_CSA, pectoralis minor cross-sectional area; SEM, standard error of measurement.

**Table 3:** Between-day intra-rater reliability in the reliability study.

Outcomes	ICC (95% CI)	SEM (mm)	SEM (%)	MDC (mm)	MDC (%)
Pm_L (mm)	0.963 (0.907–0.985)	2.33	2.0	6.45	5.6
Pm_T (mm)	0.933 (0.807–0.975)	0.58	5.9	1.61	16.4
Pm_CSA (mm^2^)	0.983 (0.958–0.994)	1.83	3.7	5.08	10.34

Abbreviations: 95% CI, 95% confidence interval of ICC; ATD, acromion-to-table distance; ICC, intraclass correlation coefficient; MDC, minimal detectable change; Pm_L, pectoralis minor length; Pm_T, pectoralis minor thickness; Pm_CSA, pectoralis minor cross-sectional area; SEM, standard error of measurement.

**Table 4:** Within-day inter-rater reliability in the reliability study.

Outcomes	ICC (95% CI)	SEM (mm)	SEM (%)	MDC (mm)	MDC (%)
Pm_L (mm)	0.980 (0.950–0.992)	1.85	1.6	5.12	4.5
Pm_T (mm)	0.954 (0.885–0.982)	0.44	4.7	1.22	13.1
Pm_CSA (mm^2^)	0.982 (0.954–0.993)	1.53	3.1	4.373	8.6

Abbreviations: 95% CI, 95% confidence interval of ICC; ATD, acromion-to-table distance; ICC, intraclass correlation coefficient; MDC, minimal detectable change; Pm_L, pectoralis minor length; Pm_T, pectoralis minor thickness; Pm_CSA, pectoralis minor cross-sectional area; SEM, standard error of measurement.

## Data Availability

The data supporting the findings of this study are not publicly available due to privacy and ethical restrictions.

## Abbreviations

The following abbreviations are used in this manuscript:

Pm_L	Pectoralis minor length
Pm_T	Pectoralis minor thickness
Pm_CSA	Pectoralis minor cross-sectional area
Pm	Pectoralis minor
ICC	Intraclass correlation coefficient
SEM	Standard errors of measurement
MDC	Minimal detectable change
ATD	Acromion-to-table distance
MRI	Magnetic resonance imaging
USI	Ultrasonography
